# Deep Power Vision Technology and Intelligent Vision Sensors

**DOI:** 10.3390/s23249626

**Published:** 2023-12-05

**Authors:** Ke Zhang, Yincheng Qi

**Affiliations:** Department of Electronic & Communication Engineering, School of Electrical and Electronic Engineering, North China Electric Power University, 619 Yonghuabei Dajie, Baoding 071000, China; qiych@ncepu.edu.cn

## 1. Introduction

With the rapid development of the power system and the increasing burden of its inspection, more attention has been paid to the automatic inspection technologies based on deep power vision technology and intelligent vision sensors. Deep power vision technology is aiming at processing and analyzing the inspection images and videos obtained through vision sensors on unmanned aerial vehicles or robots with deep learning-based computer vision algorithms. In the latest research, deep power vision technology has been widely used in the scene of processing the goals and defects of power plants, transmission lines, substations, and distribution lines in electric power systems.

## 2. Overview of Contribution

This Special Issue aims to provide some up-to-date solutions to the problems of inspection in the power system and offer helpful reference for further research of deep power vision technology and intelligent vision sensors. It includes ten papers covering the tasks of detection for the inspection of transmission lines and substation [1,2,3,4,5,6,7], classification related to the requirements of inspection [8,9], and image defogging for transmission lines [10].

To achieve a better performance in the detection tasks of the transmission line, the following methods were proposed. Wang et al. [1] proposed a fitting detection method based on multi-scale geometric transformation and an attention-masking mechanism, which demonstrated its effectiveness in improving the detection accuracy of transmission line fittings. In order to solve the problem of background interference and overlap caused by the axis-aligned bounding boxes in the tilting insulator detection tasks, Zhao et al. [2] designed a normal orientation detection method incorporating the angle regression and a priori constraints. In terms of restraining the negative impact of the large-scale gap of the fittings in the transmission line inspection, Zhao et al. [3] developed an optimized method based on contextual information enhancement and joint heterogeneous representation. Han et al. [4] were concerned with the shortcomings of IoU as well as the sensitivity of small targets to the model regression accuracy and proposed an improved YOLOX to solve the problem of low accuracy of insulator defect detection. Zhai et al. [5] introduced a multi-geometric reasoning network to accurately detect insulator geometric defects based on aerial images with complex backgrounds and different scales which significantly improved the detection accuracy of multiple insulator defects using aerial images. Xin et al. [6] combined the defogging algorithm with a two-stage detection model in order to accomplish the accurate detection of the insulator umbrella disc shedding in foggy weather. And for better inspection in a substation, Li et al. [7] presented a two-level defect detection model for alleviating the adverse effect of the complex background of substation in the infrared images.

In order to explore the solution of classification problems related to the requirements of inspection, two research articles were included in this issue. Since there are always limited defect data existing in the power system, Wang et al. [8] incorporated a semantic information fusion method based on matrix decomposition and a spatial attention mechanism to improve the classification accuracy for unseen images. For accurate identification of the bolt defect, Liu et al. [9] proposed a bolt defect identification method in which an attention mechanism and wide residual networks were combined.

This Issue also contains a method of image defogging under the scene of transmission line inspection. In terms of the fuzziness and the concealment problems in inspection images caused by fogs, Zai et al. [10] created the UAV-HAZE dataset for power assessment of unmanned aerial vehicles and presented a dual attention level feature fusion multi-patch hierarchical network for single-image defogging.

## 3. Conclusions

In conclusion, though a wide range of solutions to a variety of problems in the inspection of power system are presented in this Special Issue, the investigation of deep power vision technology and intelligent vision sensors still has a long way to go. We hope this Special Issue will provide some inspiration to researchers or engineers from academic or industrial backgrounds and further boost the fundamental and practical research in this direction.

## References

[1] Wang N., Zhang K., Zhu J., Zhao L., Huang Z., Wen X., Zhang Y., Lou W. (2023). Fittings Detection Method Based on Multi-Scale Geometric Transformation and Attention-Masking Mechanism. Sensors.

[2] Zhao J., Liu L., Chen Z., Ji Y., Feng H. (2022). A New Orientation Detection Method for Tilting Insulators Incorporating Angle Regression and Priori Constraints. Sensors.

[3] Zhao L., Liu C., Qu H. (2022). Transmission Line Object Detection Method Based on Contextual Information Enhancement and Joint Heterogeneous Representation. Sensors.

[4] Han G., Li T., Li Q., Zhao F., Zhang M., Wang R., Yuan Q., Liu K., Qin L. (2022). Improved Algorithm for Insulator and Its Defect Detection Based on YOLOX. Sensors.

[5] Zhai Y., Hu Z., Wang Q., Yang Q., Yang K. (2022). Multi-Geometric Reasoning Network for Insulator Defect Detection of Electric Transmission Lines. Sensors.

[6] Xin R., Chen X., Wu J., Yang K., Wang X., Zhai Y. (2022). Insulator Umbrella Disc Shedding Detection in Foggy Weather. Sensors.

[7] Li B., Wang T., Hu Z., Yuan C., Zhai Y. (2022). Two-Level Model for Detecting Substation Defects from Infrared Images. Sensors.

[8] Wang Y., Feng L., Song X., Xu D., Zhai Y. (2023). Zero-Shot Image Classification Method Based on Attention Mechanism and Semantic Information Fusion. Sensors.

[9] Liu L., Zhao J., Chen Z., Zhao B., Ji Y. (2022). A New Bolt Defect Identification Method Incorporating Attention Mechanism and Wide Residual Networks. Sensors.

[10] Zai W., Yan L. (2023). Multi-Patch Hierarchical Transmission Channel Image Dehazing Network Based on Dual Attention Level Feature Fusion. Sensors.

